# Validation and Comparative Analysis of a Contemporary Non-Contact Corneal Aesthesiometer

**DOI:** 10.3390/jcm15083145

**Published:** 2026-04-20

**Authors:** Ally L. Xue, Alexis Ceecee Britten-Jones, Dian Zhuang, Catherine J. Jennings, Alex Muntz, Stuti L. Misra, Laura E. Downie, Jennifer P. Craig

**Affiliations:** 1 Department of Ophthalmology, Aotearoa New Zealand National Eye Centre, The University of Auckland, Auckland 1023, New Zealandc.jennings@auckland.ac.nz (C.J.J.); s.misra@auckland.ac.nz (S.L.M.); 2 Department of Optometry and Vision Sciences, University of Melbourne, Parkville 3053, Australia; 3 Institute of Optometry, University of Applied Sciences and Arts Northwestern Switzerland, 4600 Olten, Switzerland

**Keywords:** corneal sensitivity threshold, instrument comparability, non-contact corneal aesthesiometer, ocular surface sensation

## Abstract

**Background**: Corneal sensitivity is a key indicator of ocular surface health. This prospective, cross-sectional study evaluated agreement between corneal sensitivity thresholds obtained from equivalent stimulus settings on a contemporary, enhanced dual-temperature non-contact corneal aesthesiometer (NCCA) and a previously validated (standard) device. **Methods**: Central corneal sensitivity thresholds were measured in the right eyes of healthy participants using both devices. Participants with previous ocular surgery, laser treatment, trauma, contact lens wear, diabetes, or peripheral neuropathy were excluded. Sensitivity thresholds were determined using a forced-response, double-staircase protocol. Inter-device agreement was assessed using Bland–Altman analysis, and consistency was assessed using intraclass correlation coefficients. **Results**: Median corneal sensitivity thresholds in 51 healthy participants (32 female, 19 male; mean age: 33 ± 14 years) did not differ between enhanced (0.23 [0.18 to 0.38]) and standard (0.25 [0.15 to 0.35]) NCCA instruments (*p* = 0.73). Bland–Altman analysis demonstrated moderate inter-device agreement, with a mean difference of −0.01 mbar (95% limits of agreement: −0.41 to 0.39 mbar). Linear regression analysis identified greater measurement discrepancies at higher thresholds (*p* < 0.05), indicating greater variability in individuals with reduced corneal sensitivity. **Conclusions**: The enhanced NCCA yields reliable corneal sensitivity measures for a room-temperature stimulus and acceptable agreement with the existing (standard) model.

## 1. Introduction

The cornea is the most densely innervated tissue of the human body [1,2], with a rich sensory network essential for maintaining ocular surface function [3]. This network comprises three receptor subtypes, each responding to distinct stimuli: polymodal nociceptors, which detect thermal, chemical, and mechanical stimuli; cold thermoreceptors, which respond to cooling sensations and tear osmolarity changes; and mechano-nociceptors, which are activated exclusively by mechanical stimuli [4]. These receptors play a crucial role in tear film regulation [5], detecting foreign bodies or noxious stimuli [6], and providing neurotrophic support to the corneal epithelium [3].

Corneal sensitivity is an important indicator of ocular surface health, especially in conditions where sensory nerve function may be altered, such as dry eye disease (DED) [7,8], post-refractive surgery [9,10], contact lens wear [11,12], and systemic diseases (e.g., diabetes) that cause peripheral neuropathy [13,14]. An ability to reliably measure corneal sensitivity has thus become increasingly important for clinicians and researchers studying and monitoring such conditions.

Two primary corneal aesthesiometry techniques, contact and non-contact methods, exist. The Cochet–Bonnet aesthesiometer is a contact method that stimulates mechanosensory and polymodal neurons in the corneal epithelium [15]. It uses the tip of a retractable fine nylon filament of adjustable length, which is pressed against the cornea to induce a sensory response. The longest length of filament eliciting a sensation response is recorded as the corneal sensitivity threshold. Shorter filament lengths (i.e., greater mechanical pressure) correspond to poorer corneal sensation [16]. While the Cochet–Bonnet method is rapid, portable, and widely accessible, it is invasive and offers limited, discrete intensity ranges (with a maximum filament length of 6 cm). Seeking to address these limitations, non-contact corneal aesthesiometers (NCCAs) were developed. NCCAs direct calibrated air, liquid, or gas jet pulses at the cornea, which can induce chemical, thermal, and/or mechanical stimulation depending on the nature of the stimulus [17]. These instruments offer a less invasive, potentially more precise and scientifically robust alternative method for evaluating corneal sensory nerve function. Earlier models include the Belmonte gas aesthesiometer [18] and Murphy’s NCCA [16], with subsequent advancements leading to the development of newer devices [19,20,21,22].

In DED, physical and chemical disturbances to the cornea, resulting from abnormal tear function, can stimulate all three corneal nociceptor subtypes [23]. Recent findings from experimental dry eye models suggest that tear hyperosmolarity, within the range observed in DED, primarily stimulates cold thermoreceptors, supporting the hypothesis that increased corneal cold thermoreceptor activity contributes to ocular discomfort in affected patients [24,25,26,27,28,29]. In addition to physiological and pathological factors, variations in measured corneal sensitivity can arise from the testing instrument and the stimulus type. An understanding of inter-instrument variability is important for defining the comparability of corneal sensitivity measurements obtained in different studies.

To broaden the range and functionality of instruments available for assessing corneal sensitivity, a newly developed, enhanced model NCCA (SDZ Aesthesiometer, SDZ Electronics, Auckland, New Zealand) expanding upon an earlier validated (standard) model NCCA (SDZ Aesthesiometer, SDZ Electronics, Auckland, New Zealand) [30] was evaluated. This enhanced NCCA model additionally incorporates an inbuilt cooling mechanism, allowing air stimulus delivery at both room temperature (23–24 °C) and a reduced temperature (18–19 °C). While similar NCCA models have been validated [16,30], the inter-device agreement of the enhanced SDZ NCCA relative to the earlier (standard) model is untested. Furthermore, given that corneal sensitivity is a useful indicator of ocular surface health in DED [7], it is important to determine whether inter-device agreement is maintained across differing ocular surface conditions. Therefore, this study aimed to investigate the agreement between a new (enhanced) and an earlier, established (standard) SDZ NCCA at their room-temperature air stimulus settings, to evaluate their clinical comparability and applicability for measuring corneal sensitivity, and to examine their performance in the context of a patient’s dry eye status.

## 2. Materials and Methods

This study was conducted in compliance with the Declaration of Helsinki and received institutional ethical review board approval (UAHPEC #024360). Written informed consent was obtained from all participants. Adults (18 years and over) without known eye diseases were eligible to participate. Individuals with a history of corneal pathology, prior ocular surgery, corneal laser treatment, trauma, contact lens wear, diabetes, or any systemic condition associated with peripheral neuropathy were excluded.

Corneal sensitivity threshold assessments were conducted within a climate-controlled ophthalmic clinical site, under standard room illumination, between 09:00 and 17:00 h. It has been previously demonstrated that corneal sensitivity is not influenced by normal variations in ambient temperature or humidity, or over the course of a working day [31,32]. Ceiling vents were partitioned from the testing area to eliminate extraneous airflow that could interfere with measurement accuracy.

### 2.1. Instrumentation

The design of both the enhanced and standard NCCA models tested in the study was modified from Murphy’s NCCA model (Glasgow Caledonian University, Glasgow, UK) [16], and the instrument set-up has been described previously [30]. Instrument comparison and validation were tested only for the equivalent room-temperature air stimuli.

Each device was mounted on a slit lamp biomicroscope (Takagi SL-D701, Takagi Seiko Co., Ltd., Nakano, Japan) for precise physical alignment. The brass stimulus jet, featuring a 0.5 mm bore, delivered air stimuli ranging from 0.1 to 20 mbar over an approximate corneal area of 0.8 mm^2^. The stimulus duration was standardised at 0.9 s, with incremental pressure increases of 0.1 mbar. A built-in video camera, located inferiorly to the bore, facilitated real-time monitoring of eye movements and blinking during testing. Markers on the camera screen ensured accurate alignment of the stimulus delivery to the central cornea throughout the measurement protocol.

### 2.2. Testing Procedure

In all participants, the right eye was tested. Examinations were undertaken by one of three trained examiners (ACB-J, DZ, or CJJ) using an identical protocol, as described previously [33]. Each NCCA device was calibrated and set to zero prior to participant testing. To minimise bias and mitigate against a test-order effect, participants were randomised to undergo assessment with either the standard or enhanced NCCA first, followed by the second device after 3–5 min. The order of instrument presentation was not masked to the examiners or participants. The randomisation sequence was generated prior to study start to prevent influence by researchers involved in data collection. Central corneal sensitivity thresholds were measured, with lower threshold values indicating greater corneal sensitivity (for both NCCA devices).

Corneal sensitivity thresholds were determined using a double-staircase forced-choice protocol (where participants indicated either “felt” or “not felt” to each stimulus) [33]. To standardise stimulus presentation, an eight-second audio cue was developed and played during each trial, incorporating a white-noise background to mask surrounding noise that might interfere with responses. The start of the audio cue signalled the beginning of each trial (the presentation and evaluation of a single stimulus). The audio cues also included verbal prompts instructing participants to blink before each stimulus was delivered. In the audio cue, participants were instructed to blink several times upon hearing a short beep (at 2 s) and then to keep their eyes open for a subsequent long, continuous beep (at 5 s, lasting for 3 s) during which the stimulus was presented. Participants were instructed to blink freely between and immediately before individual stimulus presentations.

The audio cue was played to each participant through noise-cancelling headphones (Philips Active Noise-Cancelling headphones, SHL3850NC, Shenzhen, China), while the examiner was guided by a linked video to synchronise the stimulus timing. Participants were informed that a variable strength stimulus may or may not be emitted during the long continuous beep. After each trial, participants verbally responded “felt” or “not felt” immediately after the recording ended.

### 2.3. Threshold Determination

Participants were positioned comfortably at the slit lamp biomicroscope and instructed to fixate on a central target using their non-test eye. The bore tip of the NCCA was positioned 10 mm from the anterior corneal surface, aligned with the geometric centre of the cornea [30]. Limbal markers on the NCCA camera screen allowed fine adjustments to be made, to align with the temporal and nasal limbus, ensuring accurate placement of the device prior to delivery of each air stimulus.

The order of stimulus presentation followed a double-staircase algorithm written in MATLAB (MATLAB, version 9.2 (R2017a), The MathWorks Inc., Natick, MA, USA). Testing commenced with a predetermined threshold for each staircase. A suprathreshold stimulus was initially presented to familiarise participants with a detectable stimulus and establish the starting threshold. To determine the starting threshold, participants were first presented with a 0.3 mbar stimulus; if they did not feel it, the stimulus was increased in 0.3 mbar steps until detected. The lowest detectable threshold was used as the mean starting value for the algorithm, with a random jitter of 0.1 mbar (i.e., a mean starting value set at 0.3 mbar could begin between 0.2 and 0.4 mbar). A double-staircase procedure was then run with three reversals per staircase (with the first reversal ignored in determining the final threshold) and a step size of 0.1 mbar. Trials were repeated until a final threshold for the central cornea was determined. Examiners included between 1 and 3 false-positive stimuli per test. For false-positive testing, the examiner would set up the trial but not press the stimulus release button, which was obscured from the participant.

To account for the instrument’s minimum stimulus pressure (0.1 mbar), the double-staircase algorithm entered a decision tree if two consecutive stimuli were detected at the lowest possible level (Figure S1). In the decision tree, if the participant reported “felt” to two stimuli at 0.1 mbar and “not felt” to two false-positive checks, the threshold was recorded as <0.1 mbar. If the participant responded “felt” to one of the two 0.1 mbar stimuli and none of the false-positive checks, the final threshold was recorded as 0.1 mbar. If the participant responded “felt” to both false-positive checks, testing was stopped, participants were re-instructed, and the entire staircase was repeated for that measurement.

### 2.4. Ocular Surface Evaluation

Following NCCA testing, the dry eye status of the participant’s right eye was assessed with an established rapid, non-invasive clinical testing protocol [34]. The Symptom Assessment iN Dry Eye (SANDE) questionnaire allowed quantification of dry eye symptomology [35]. Ocular surface parameters were evaluated using the Oculus Keratograph 5M (K5M) (Oculus, Wetzlar, Germany) and included tear film lipid layer grade, the average of three measurements for non-invasive tear film breakup time (seconds), and tear meniscus height (mm) [34].

### 2.5. Data and Statistical Analysis

Bland–Altman plots were used to evaluate the level of agreement between corneal sensitivity threshold measurements derived from the two NCCA devices (methods). The mean difference for measures taken using the two methods was plotted against the average of the measures. The mean bias and 95% limits of agreement (LoA), which define the range within which 95% of the differences between paired measurements, are expected to fall, were also determined [36]. Visual inspection of the Bland–Altman plots demonstrated heteroscedasticity. Log transformation was explored; however, this approach reduced clinical interpretability due to the low magnitude of measurement values. Therefore, magnitude-dependent limits of agreement were derived using the residual standard deviation from the fitted model [36]. This approach allows LoA to vary across the measurement range, providing a more appropriate representation of agreement under non-uniform variability. Linear regression analysis was undertaken to investigate how measurements varied across the range of measured corneal sensitivities.

To evaluate measurement reliability between the devices, intraclass correlation coefficients (ICCs) and 95% confidence intervals were calculated, using a single-rating, absolute-agreement, two-way random effects model. ICC values were interpreted following the approach of Koo et al. [37], with ICC < 0.50 indicative of poor agreement, 0.50 ≤ ICC < 0.75 indicative of moderate agreement, 0.75 ≤ ICC < 0.90 indicative of good agreement, and ICC > 0.90 indicative of excellent agreement.

A Shapiro–Wilk test was performed to assess whether the differences between measurements were normally distributed. To compare measurements between the enhanced and standard NCCA devices, the paired t-test was utilised for normally distributed data and the Wilcoxon Signed-Rank Test, for non-normally distributed data. Given the randomised, non-blinded nature of the instrument testing order, supplementary analyses (Bland–Altman plots and appropriate paired-difference testing) were undertaken to evaluate potential device test-order effects.

Previous reports on the relationship between DED and corneal sensitivity show inconsistency, potentially influenced by corneal nerve density and morphology, as well as methodological heterogeneity [38]. To investigate the possible influence of DED on measurement variability, participants were classified by DED status, according to the prevailing global consensus Tear Film and Ocular Surface Society Dry Eye Workshop II (TFOS DEWS II) criteria, and the mean inter-instrument differences between groups were compared (two-sample t-test) to assess for any disease-related effect on measurement bias.

As this study aimed to assess the validation and comparative performance of the devices rather than test a predefined hypothesis of group differences, a formal power calculation was not conducted. A post hoc minimum detectable mean paired difference was calculated for 80% power and α = 0.05 using the observed standard deviation of paired differences and study sample size (n = 51).

Data analyses were conducted using SPSS software (version 29.0 for Windows; SPSS Inc., Chicago, IL, USA) and R Statistical Software (version 4.5; R Foundation for Statistical Computing, Vienna, Austria). Data are expressed as mean ± standard deviation (SD) or median (interquartile range, IQR), as appropriate, unless otherwise specified. Statistical significance was defined as *p* < 0.05.

## 3. Results

A total of 51 right eyes of 51 participants (32 female, 19 male) were examined. The mean age of participants was 33 ± 14 years (range: 18 to 74 years). Participant demographics and ocular surface characteristics are summarised in Table 1. Twenty-eight participants (55%) underwent testing with the standard instrument first; the remaining participants (45%) were tested with the enhanced instrument first.

Median (IQR) central corneal sensitivity thresholds (n = 51 participants) were 0.23 (0.18 to 0.38) mbar and 0.25 (0.15 to 0.35) mbar, for the enhanced and standard NCCA devices, respectively. There was no statistically significant difference in corneal sensitivity perception threshold readings between the two devices (Wilcoxon Signed-Rank Test, *p* = 0.73; Figure S2). Post hoc analysis using the observed standard deviation of paired inter-device differences and n = 51 showed that the minimum detectable mean paired difference was 0.08 mbar (80% power, α = 0.05). The median [IQR] inter-device difference in corneal sensitivity threshold was also similar between groups with (−0.025 [−0.14 to 0.1] mbar) and without DED (0 [−0.11 to 0.13] mbar; *p* = 0.11).

Bland–Altman analysis of corneal sensitivity thresholds (Figure S3) showed moderate agreement between the enhanced and standard NCCA devices, with a mean difference of −0.01 mbar (95% limits of agreement: −0.41 to 0.39 mbar). No systematic bias was observed; differences were centred around zero, indicating that neither instrument consistently over- nor under-estimated corneal sensitivity thresholds relative to the other. In addition, no systematic bias was evident in the Bland–Altman plots (Figure S4) when stratified by testing order (i.e., standard instrument first vs. enhanced instrument first). A two-sample t-test comparing inter-device differences between participants randomised to the enhanced device first (n = 23), and those randomised to the standard device first (n = 28) showed no evidence of a sequence effect (*p* = 0.70).

**Figure 1:**
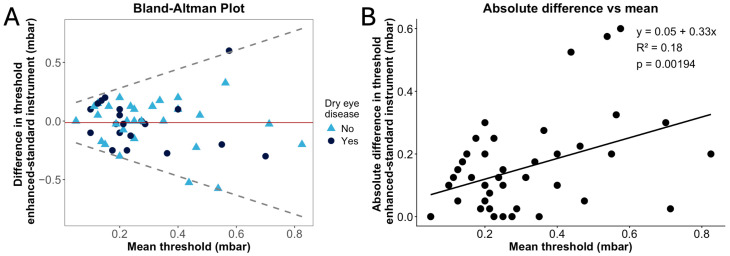
(**A**): Bland–Altman plot comparing central corneal sensitivity thresholds measured with the enhanced and standard non-contact corneal aesthesiometer (NCCA) devices using room-temperature stimuli. Solid line represents mean difference; dashed lines represent magnitude-dependent limits of agreement (LoA) derived from modelling the relationship between absolute differences and the mean, accounting for heteroscedasticity. “Yes” and “No” labels indicate participant dry eye disease status. Some points are coincident, resulting in fewer than 51 visible plot points. (**B**): Linear regression showing that the difference between threshold measures from the enhanced and standard NCCA devices was greater when the central corneal sensitivity threshold was higher (i.e., the cornea was less sensitive).

However, visual inspection of the Bland–Altman plot (Figure S3) showed increasing variability in the differences between the two instruments at higher mean thresholds, suggesting proportional heteroscedasticity. To account for this, magnitude-dependent limits of agreement were modelled to reflect this relationship (Figure 1A) [36]. In addition, linear regression was performed to examine the relationship between absolute inter-device difference and mean threshold across the measurement range (Figure 1B). The regression model demonstrated that the absolute inter-device difference in threshold increased by 0.33 mbar for every 1 mbar increase in mean threshold (*p* < 0.05).

**Figure 2:**
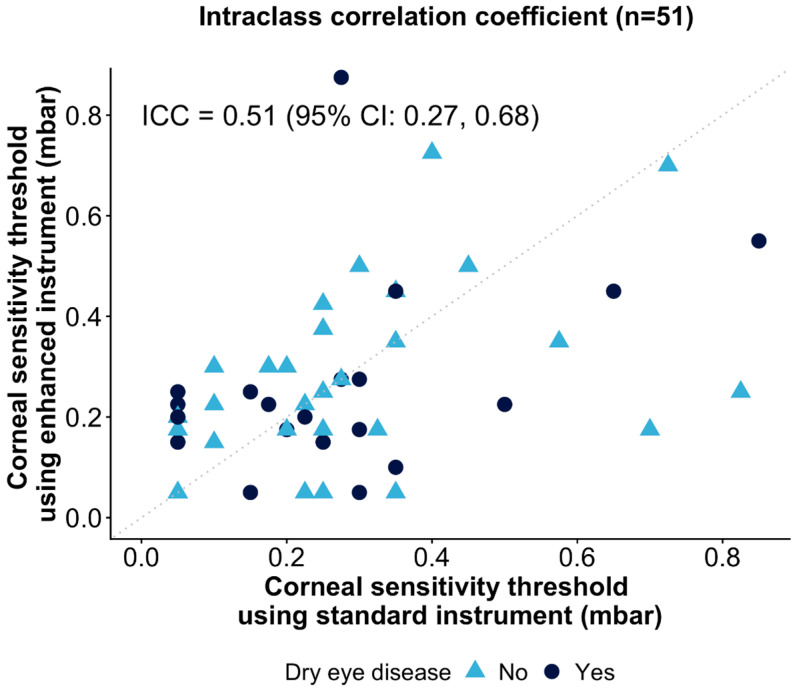
Intraclass correlation coefficient (ICC) between the enhanced and standard NCCA devices for all participants (n = 51). The ICC was calculated using a two-way model with absolute agreement for single measurements. The dotted line shows the line of identity (perfect agreement). “Yes” and “No” labels indicate participant dry eye disease status.

Intraclass correlation coefficient (ICC) analysis (Figure 2) demonstrated moderate agreement between the two NCCA devices (ICC = 0.51 [95% CI: 0.27, 0.68]). Additional ICC analyses on participant subgroups separated by dry eye status yielded similarly moderate agreement in participants with DED (ICC = 0.44 [95% CI: −0.01, 0.74]) and without DED (ICC = 0.55 [95% CI: 0.24, 0.75]).

## 4. Discussion

Corneal sensitivity is an important clinical parameter for assessing ocular surface health, including in DED [7,38,41]. Recent studies suggest that tear hyperosmolarity associated with DED may primarily excite corneal cold thermoreceptors, contributing to ocular discomfort in affected patients [24,25,26,27,28,29]. A newly developed (enhanced) model NCCA (SDZ Aesthesiometer, SDZ Electronics, Auckland, New Zealand) was designed based on an earlier validated (standard) model [30] to broaden the range and functionality of instruments available for assessing corneal sensitivity.

The present study evaluated the agreement between central corneal sensitivity measures quantified using the enhanced NCCA device, relative to an established standard NCCA instrument, for air stimuli administered at room temperature. The ICC between the two instruments was 0.51 (95% CI: 0.27, 0.68), indicating moderate reliability. Bland–Altman analysis showed a mean bias of −0.01 mbar, with no significant systematic bias, indicating that neither instrument consistently produced higher (or lower) values than the other. Swanevelder et al. [30] previously analysed inter-device measurement differences between the established Murphy’s NCCA prototype [42] and the standard NCCA evaluated in the present study, and reported good intra-observer agreement, with six data points lying at (0,0) on the Bland–Altman plot. Together, these data highlight the importance of defining inter-instrument performance and, thus, measurement comparability across devices, particularly in the context of designing multicentre and longitudinal clinical studies where repeated measures are taken.

In addition, Bland–Altman analysis demonstrated heteroscedasticity, and linear regression analysis confirmed magnitude-dependent measurement variability, with higher inter-instrument differences at higher sensitivity thresholds (i.e., in cases of poorer corneal sensitivity). Similar heteroscedasticity was reported by Nosch et al., who found that inter-instrument agreement between a novel NCCA and the Cochet–Bonnet aesthesiometer was considerably better in younger participants than in an older group exhibiting overall higher sensitivity thresholds [22]. In other ophthalmic contexts that adopt psychophysical threshold-based diagnostic tools, such as visual field perimetry, test–retest variability is well documented to increase as measured sensitivity decreases [43]. Therefore, in the context of the present study, it is not unexpected that reduced corneal sensitivity is accompanied by increased sensory noise, which likely contributes to the greater variability observed among participants with poorer threshold measurements. This insight should be considered when interpreting corneal sensitivity findings in a higher threshold range, although no values exceeded 1 mbar in this study. Nevertheless, these findings suggest that more pronounced differences in quantified corneal sensitivity may occur if the two devices are used to quantify thresholds in individuals with corneal sensory nerve pathology. Given existing evidence that corneal sensitivity thresholds may be more variable in ocular surface disease states, such as DED [7], future studies should examine variability in disease-specific populations to better understand how corneal status may affect the reliability of corneal sensitivity measurements.

Although the two tested NCCA devices are built on the same principles, the devices showed inter-device limits of agreement up to 0.40 mbar. There is no universally recognised normative value for central corneal sensitivity. Reported values in healthy (“normal”) eyes range from 0.1 to 6 mbar and are instrument-dependent [8,19,33,42], with measurements from non-contact methods not directly comparable to those of the mechanical Cochet–Bonnet aesthesiometer [19,32,38]. While the magnitude of the inter-device difference might be considered small in absolute terms, relative to the full reported range of “normal” corneal sensitivity values, a previous study found a mean difference of 0.30 mbar between healthy individuals and individuals with diabetes and subclinical neuropathy using the enhanced NCCA device [33]. Exchanging instruments could, therefore, potentially obscure clinically important small differences, such as early or subclinical corneal neuropathy.

While this study investigated two SDZ NCCA devices, both based on the Murphy NCCA instrument, the observed differences between the enhanced and standard NCCAs highlight the need to consider inter-instrument variability when comparing corneal sensitivity data quantified using different NCCA devices. Using the same instrument is therefore advisable for longitudinal assessment. Future studies comparing these NCCA devices with other instruments, such as the Belmonte NCCA [18] and commercially available Brill Esthesiometer [20], would be beneficial for developing a more informed understanding of cross-study comparability of corneal sensitivity threshold measures in both health and disease.

The enhanced NCCA introduces extended functionality, with the addition of a cooled air jet stream, offering novel diagnostic potential in conditions in which corneal TRPM8 cold receptor subtypes may be more severely affected, such as in diabetes [44]. Its ease of use for clinical settings further enhances its potential utility. The enhanced NCCA was shown in the present study to provide useful, direct assessment of corneal sensory function, supporting its application not only in ophthalmology, but potentially in broader fields such as neurophysiology and endocrinology, where objective assessments of nerve function can be useful.

While findings from this study are promising, certain limitations are acknowledged. An a priori power calculation was not performed as this study aimed to compare devices rather than to test a prespecified difference; however, we computed a post hoc minimal detectable mean paired difference based on the observed inter-device differences in corneal sensitivity perception threshold readings to inform sample size planning for future studies. Larger, more heterogeneous samples (for example, including individuals with established corneal neuropathy) are needed to evaluate inter-device agreement across a broader range of corneal sensitivity values. Furthermore, we assessed DED status to examine any potential impact on measurement variability; however, this study was not specifically powered to detect differences between participants with and without DED, and larger studies encompassing participants exhibiting a broader range of DED subtypes and severity are required to address this question. In addition, while the current study aimed to evaluate inter-device comparability, there remains a need to investigate test–retest variability of the NCCA device in future studies.

Measurements were also restricted to a single central corneal location for consistency in X-Y alignment and to facilitate pulse delivery perpendicular to the corneal surface, but corneal sensitivity is acknowledged to vary across different corneal regions [45,46] and it is possible that inter-device measurement agreement could differ in other corneal locations. Future studies could aim to incorporate testing at multiple corneal eccentricities to more comprehensively define inter-instrument measurement comparability.

Inter-observer reproducibility and intra-observer agreement were also not evaluated in this study; an understanding of these parameters would be useful for fully defining the enhanced NCCA instrument’s research utility. Nevertheless, this study achieved its primary objective: to define the agreement of central corneal sensitivity threshold measurements obtained using a contemporary, enhanced NCCA compared to a previously validated (standard) device. Future research can build upon these findings by including broader participant demographics and clinical characteristics, to further understand device performance in different clinical scenarios.

## 5. Conclusions

In conclusion, this study demonstrates a moderate agreement between central corneal sensitivity measures to room air temperature stimuli derived from an enhanced NCCA, one of few non-contact devices with the potential to administer a cooled air jet stimulus, and a predecessor (standard) NCCA device. Moderate agreement was observed across participants with varying ocular surface status, including those with DED. These findings suggest that measurements taken on the two NCCA devices are not necessarily interchangeable and highlight the importance of considering inter-device variability. Both the standard and enhanced NCCA devices are portable, non-invasive tools for assessing corneal sensitivity, making them well-suited to clinical and research settings. The added ability to deliver cooled stimuli with the enhanced NCCA may expand its application to a range of ocular diseases, particularly for conditions that affect corneal cold thermoreceptors. With a stimulus interval of 0.1 mbar, and a measurable range of 0.1 to 20 mbar, both instruments allow for finer stimulus intensity adjustments than similar commercially available instrumentation that offers five discrete stimulus levels and a pressure range of 2 to 10 mbar [47], potentially enabling detection of more subtle differences in corneal sensitivity.

## Figures and Tables

**Table 1:** Participant (n = 51) demographic and ocular surface characteristics. Summary statistics are presented as mean ± SD for parametric data, median (IQR) for non-parametric data.

	Characteristic	Values
**Demographics**	Age (years)	33 ± 14
	Female gender [%]	32 [62.7%]
**Dry eye symptomology**	SANDE score (out of 100)	29 (14–49)
**Tear film measures**	Non-invasive tear film breakup time (s)	5.65 (3.76–8.75)
	Lipid layer grade (modified Guillon-Keeler grading, out of 5)	3 (3–4)
	Tear meniscus height (mm)	0.24 (0.19–0.29)
**Dry eye disease classification ^a^**	Overall diagnosis of dry eye disease [%]	20 [39.2%]
	Lipid-deficient evaporative dry eye signs ^b^	12 [23.5%]
	Aqueous-deficient dry eye signs ^b^	8 [15.7%]

^a^ Dry eye disease was diagnosed using an abridged, non-invasive dry eye assessment algorithm based on the TFOS DEWS II criteria [34], defined by a SANDE score ≥ 30 and a non-invasive tear film breakup time < 10 s. ^b^ For subtype classification, lipid-deficient dry eye was additionally defined as a lipid layer grade of 0–3 (modified Guillon-Keeler grading, out of 5) [39], while aqueous-deficient dry eye was defined by a tear meniscus height < 0.2 mm [34,40].

## Data Availability

The data presented in this study are available on request from the corresponding author due to privacy, legal, and ethical reasons.

## Supplementary Materials

The following supporting information can be downloaded at https://www.mdpi.com/article/10.3390/jcm15083145/s1, Figure S1: Example of a non-contact corneal aesthesiometry stimulus testing protocol, in the case of two consecutive responses being detected at 0.1 mbar. Figure S2: Comparison of corneal sensitivity threshold measurements from the enhanced and standard non-contact corneal aesthesiometers. Statistical significance between instruments was assessed using a two-sided Wilcoxon Signed-Rank Test; NS indicates not significant (*p* > 0.05). Figure S3: Bland–Altman plot comparing central corneal sensitivity thresholds measured with the enhanced and standard non-contact corneal aesthesiometer (NCCA) devices using room-temperature stimuli. LoA: 95% limits of agreement. “Yes” and “No” labels indicate participant dry eye disease status. Some points are coincident, resulting in fewer than 51 visible plot points. Figure S4: Bland–Altman plot comparing central corneal sensitivity thresholds measured with the enhanced and standard NCCA devices using room-temperature stimuli, separated by testing order. (A) The enhanced device was tested first (n = 23); (B) the standard device was tested first (n = 28). LoA: 95% limits of agreement. Some points are coincident, resulting in fewer than 23 visible plot points in (A) and 28 visible plot points in (B).

## Abbreviations

The following abbreviations are used in this manuscript:

CI	Confidence interval
DED	Dry eye disease
ICC	Intraclass correlation coefficient
IQR	Interquartile range
K5M	Oculus Keratograph 5M
LoA	Limits of agreement
mm	Millimetre(s)
mbar	Millibar(s)
NCCA	Non-contact corneal aesthesiometer
SD	Standard deviation
SPSS	Statistical Package for Social Sciences
s	Seconds
TFOS DEWS II	Tear Film & Ocular Surface Society Dry Eye Workshop II
UAHPEC	University of Auckland Human Participants Ethics Committee
