# Supplementary material for: Validation and Comparative Analysis of a Contemporary Non-Contact Corneal Aesthesiometer
Source: J Clin Med. 2026 Apr 20;15(8):3145. doi: 10.3390/jcm15083145 (PMC13117072; doi:10.3390/jcm15083145)
Supplement: Supplementary file 1 [file jcm-15-03145-s001.zip › jcm-4203511-supplementary.pdf]

Supplementary Materials:

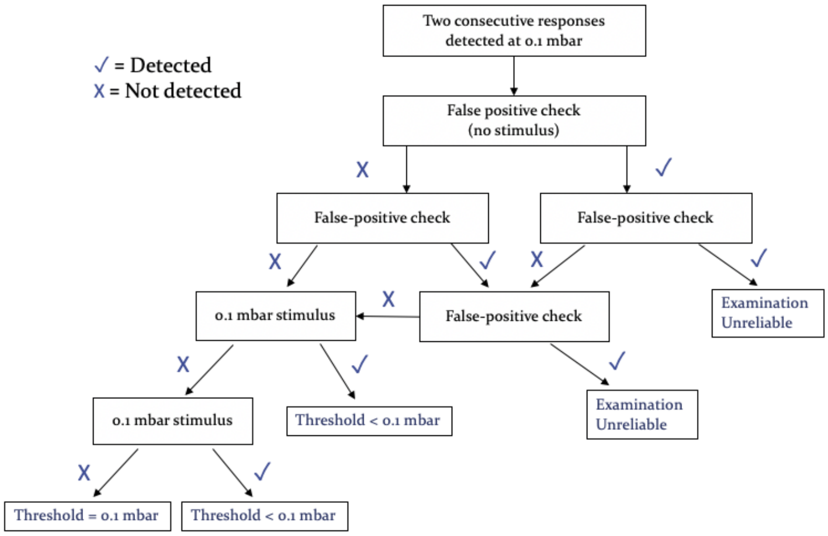

Figure S1: Example of a non-contact corneal aesthesiometry stimulus testing protocol, in the case of two consecutive responses being detected at 0.1 mbar.

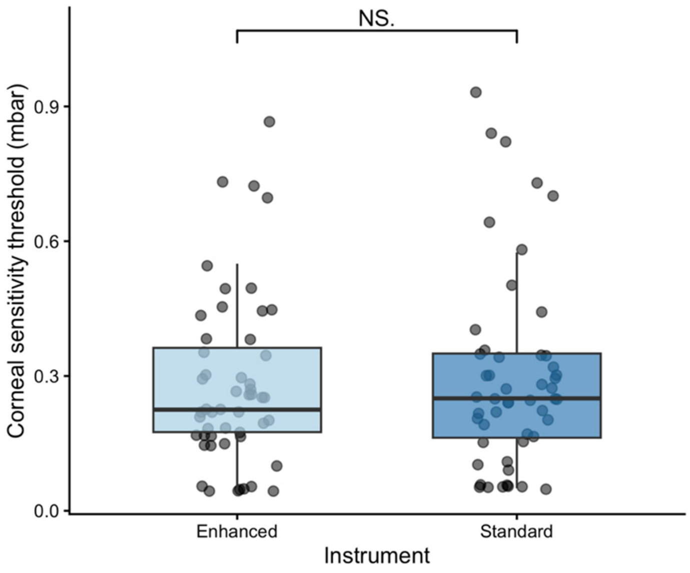

Figure S2: Comparison of corneal sensitivity threshold measurements from the enhanced and standard non-contact corneal aesthesiometers. Statistical significance between instruments was assessed using a two-sided Wilcoxon Signed-Rank test; NS indicates not significant ($p > 0.05$).

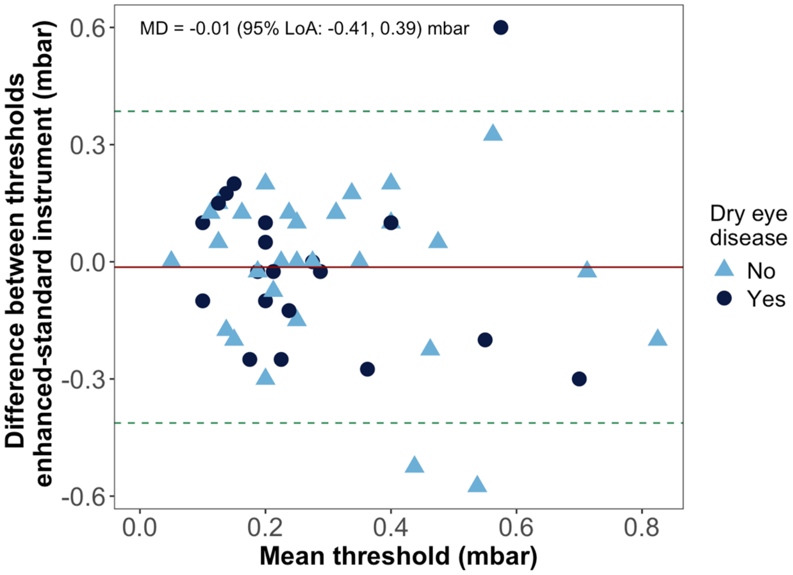

Figure S3: Bland-Altman plot comparing central corneal sensitivity thresholds measured with the enhanced and standard non-contact corneal aesthesiometer (NCCA) devices using room-temperature stimuli. LoA: 95% limits of agreement. "Yes" and "No" labels indicate participant dry eye disease status. Some points are coincident, resulting in fewer than 51 visible plot points.

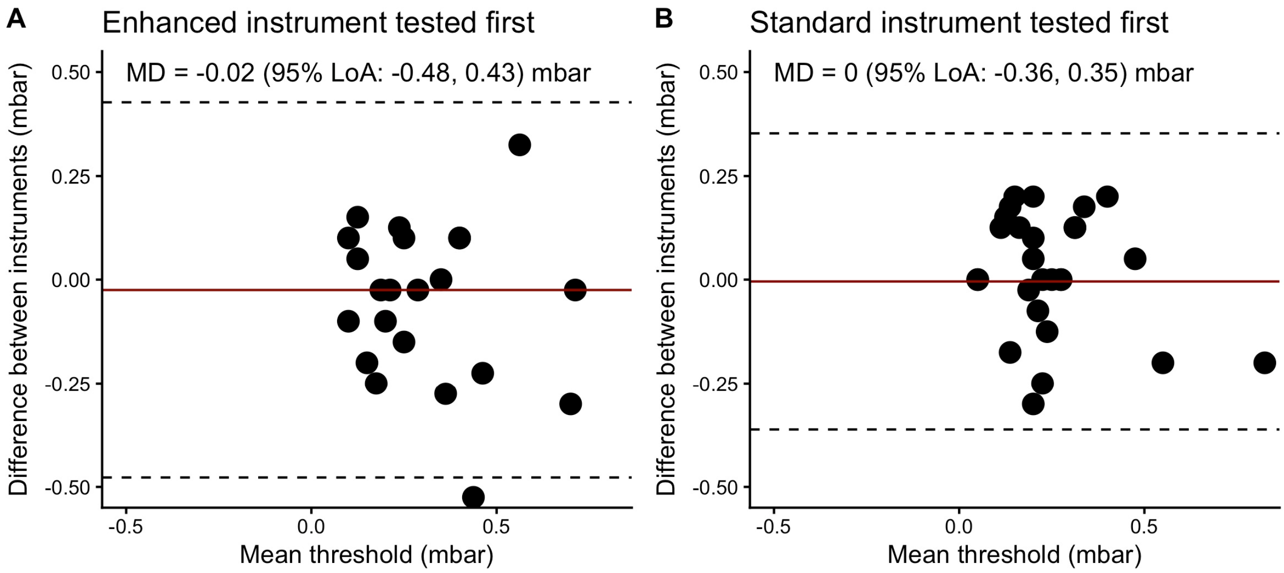

Figure S4. Bland-Altman plot comparing central corneal sensitivity thresholds measured with the enhanced and standard NCCA devices using room-temperature stimuli, separated by testing order. (A) the enhanced device was tested first (n = 23); (B) the standard device was tested first (n = 28). LoA: 95% limits of agreement. Some points are coincident, resulting in fewer than 23 visible plot points in (A) and 28 visible plot points in (B).
